# Patient similarity and other artificial intelligence machine learning algorithms in clinical decision aid for shared decision-making in the Prevention of Cardiovascular Toxicity (PACT): a feasibility trial design

**DOI:** 10.1186/s40959-022-00151-0

**Published:** 2023-01-23

**Authors:** Sherry-Ann Brown, Brian Y. Chung, Krishna Doshi, Abdulaziz Hamid, Erin Pederson, Ragasnehith Maddula, Allen Hanna, Indrajit Choudhuri, Rodney Sparapani, Mehri Bagheri Mohamadi Pour, Jun Zhang, Anai N. Kothari, Patrick Collier, Pedro Caraballo, Peter Noseworthy, Adelaide Arruda-Olson, for the Cardio-Oncology Artificial Intelligence Informatics and Precision Equity (CAIPE) Research Team Investigators

**Affiliations:** 1 Cardio-Oncology Program, Division of Cardiovascular Medicine, Medical College of Wisconsin, Milwaukee, WI USA; 2 Department of Cardiovascular Medicine, Mayo Clinic, Rochester, MN USA; 3 Cancer Center, Medical College of Wisconsin, Milwaukee, WI USA; 4 Department of Internal Medicine, Advocate Lutheran General Hospital, Park Ridge, IL USA; 5 Medical College of Wisconsin, Milwaukee, WI USA; 6 University of Wisconsin-Milwaukee, Milwaukee, WI USA; 7 Department of Electrophysiology, Froedtert South, Kenosha, WI USA; 8 Institute for Health and Equity, Medical College of Wisconsin, Milwaukee, WI USA; 9 Department of Computer Science, University of Wisconsin-Milwaukee, Milwaukee, WI USA; 10 Division of Surgical Oncology, Department of Surgery, Medical College of Wisconsin, Milwaukee, WI USA; 11 Department of Cardiovascular Medicine, Cleveland Clinic, Cleveland, OH USA; 12 Department of Medicine, Mayo Clinic, Rochester, MN USA

**Keywords:** Cardio-oncology, Cardiotoxicity, Cancer survivors, Machine learning, Artificial intelligence, Clinical decision aid, Clinical decision support

## Abstract

**Background:**

The many improvements in cancer therapies have led to an increased number of survivors, which comes with a greater risk of consequent/subsequent cardiovascular disease. Identifying effective management strategies that can mitigate this risk of cardiovascular complications is vital. Therefore, developing computer-driven and personalized clinical decision aid interventions that can provide early detection of patients at risk, stratify that risk, and recommend specific cardio-oncology management guidelines and expert consensus recommendations is critically important.

**Objectives:**

To assess the feasibility, acceptability, and utility of the use of an artificial intelligence (AI)-powered clinical decision aid tool in shared decision making between the cancer survivor patient and the cardiologist regarding prevention of cardiovascular disease.

**Design:**

This is a single-center, double-arm, open-label, randomized interventional feasibility study. Our cardio-oncology cohort of > 4000 individuals from our Clinical Research Data Warehouse will be queried to identify at least 200 adult cancer survivors who meet the eligibility criteria. Study participants will be randomized into either the Clinical Decision Aid Group (where patients will use the clinical decision aid in addition to current practice) or the Control Group (current practice). The primary endpoint of this study is to assess for each patient encounter whether cardiovascular medications and imaging pursued were consistent with current medical society recommendations. Additionally, the perceptions of using the clinical decision tool will be evaluated based on patient and physician feedback through surveys and focus groups.

**Summary:**

This trial will determine whether a clinical decision aid tool improves cancer survivors’ medication use and imaging surveillance recommendations aligned with current medical guidelines.

**Trial registration:**

ClinicalTrials.Gov Identifier: NCT05377320

## Introduction

Cardiovascular disease is a leading cause of death in cancer patients, second only to the development of recurrent or secondary cancer. Approximately two million new cancer diagnoses and > 600,000 new cancer deaths are estimated to occur each year [1]. Nearly 17 million Americans are cancer survivors, and this number is expected to increase to more than 22 million by 2030 [2, 3]. Of these 17 million survivors, four million have had breast cancer, which is often the most common cancer with treatment leading to cardiovascular complications in cardio-oncology clinics [3–9]. Improvement in cancer therapies has increased the number of patients surviving cancer, and often confer increased cardiovascular disease risk. Moreover, specific cancer therapeutic strategies have cardiovascular toxic effects, including those that employ anthracyclines and HER2 inhibitors [10]. Given the growing numbers in cancer survivors and their increased susceptibility to cardiovascular disease, a clinical decision aid intervention that classifies cancer patients most inclined to develop cardiovascular disease and identifies effective treatment strategies that mitigate cardiovascular disease progression is urgently needed.

Many cardiologists are not specifically trained to care for cancer patients and have limited familiarity with cardio-oncology recommendations [6, 11–17]. Furthermore, management and follow-up of cardiovascular risk for cardio-oncology patients and survivors often differ from that of individuals in the general population with similar cardiovascular risk factors. For example, breast cancer patients at risk for cardiovascular complications are often insufficiently treated with cardioprotective medications and appropriate frequencies of cardiac surveillance imaging [18–20]. Several cardio-oncology guidelines, medical society scientific statements, and consensus recommendations have become available in recent years [21–28]. Yet, we are challenged with how best to support physicians and cancer survivors to implement these recommendations, especially those at highest risk for developing cardiovascular disease. The relative absence of training in cardio-oncology has therefore resulted in a tremendous knowledge gap in optimal patient care [11–15].

**Fig. 1:**
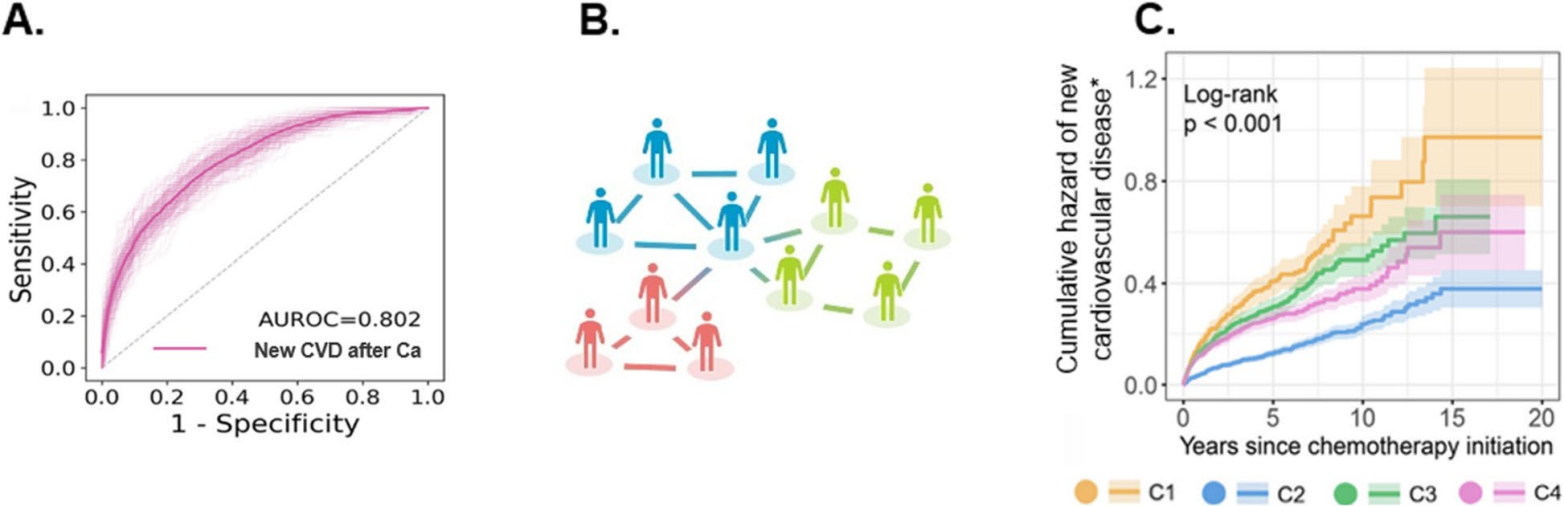
Patient Similarity Machine Learning and Network Analysis. **A** Area under receiver operator curve (AUROC; AUC) for prediction of cardiovascular diseases after cancer (Ca) diagnosis in > 4000 patients. **B** Patients most similar to each other are closest to each other and form clusters in networks; patients in the same cluster or similarity network tend to display similar rates of cardiovascular disease (CVD) and mortality. **C** Cumulative hazard of new (or de novo) CVD after cancer diagnosis; cardiovascular disease risk clusters (C1-C4) based on the patient similarity machine learning and network analysis. Used with permission [29]

A clinical decision aid powered by artificial intelligence capable of appropriately evaluating and assessing cardiovascular disease risk with evidence-based suggestions for care for cancer survivors may help bridge this gap. Notably, artificial intelligence algorithms trained on 20-year follow-up data for > 4,000 racially diverse survivors of various cancers were recently developed and validated. The algorithms use cardiovascular imaging (echocardiograms) and clinical variables and can predict cardiovascular disease events [29]. Further, some of these machine learning and network algorithms have been used to precisely predict cardiac risk assessment in a database study, by analyzing how similar various patients are to each other [29] (Fig. 1). In general, such “patient similarity” machine learning and network algorithms represent each patient as a combined vector of features and characteristics, and the similarity between two patients can be measured by a variety of distance measures. In the absence of substantial missing data, the algorithms can be used to form clusters or groups of patients to further facilitate prediction and classification. However, none of these algorithms have yet been incorporated into a clinical decision aid or used in clinical practice for cardio-oncology patients.

This trial design manuscript describes our study, in which these machine learning algorithms will be incorporated into a clinical decision aid and used in clinical practice for cardio-oncology patients. In a cohort of cancer survivors, we propose to evaluate the feasibility of this novel clinical decision aid with the expectation that it will ultimately improve medication use and imaging utilization to mitigate cardiovascular risk. For this feasibility study, we will create the clinical decision aid and test the central hypothesis that this novel clinical decision aid accessed via the electronic health record will be acceptable to both cancer survivors and their cardiologists, and will favorably impact appropriate medication use and cardiac imaging surveillance.

## Methods

### Study design

**Fig. 2:**
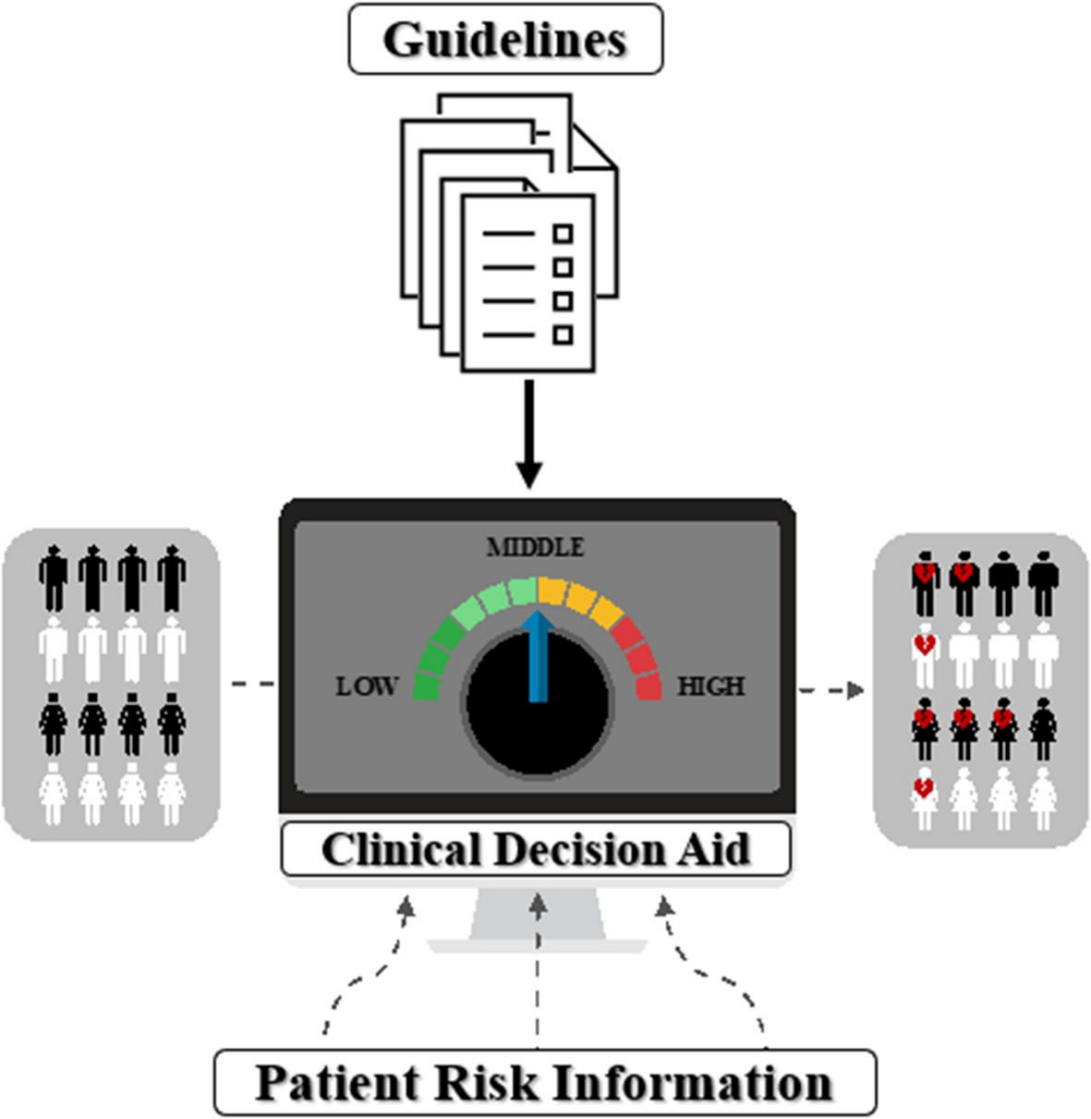
Clinical Decision Aid Incorporates Artificial Intelligence and Rules-Based Algorithms for Risk Prediction (Artificial Intelligence-Powered Personalization) and Guidelines Implementation (Rules-Based Personalization). Templates from Infograpia were used in the making of this graphic

This is a single-center, double-arm, open-label, randomized interventional feasibility study that will determine whether a novel clinical decision aid accessed via the electronic health record will be acceptable to both cancer survivors (Table 1) and their cardiologists. In the study, we will assess whether use of the tool in shared decision-making favorably impacts appropriate medication use and cardiac imaging surveillance (Table 2). The overall process for this study is as follows. Cardiovascular risk assessment will begin with the artificial intelligence algorithm based on clinical, laboratory, and echocardiographic data (Table 3). The artificial intelligence algorithms used are explainable. A separate rules-based algorithm based on existing guidelines, expert recommendations, and medical society scientific statements has also been created (Brown et al., in review) (Fig. 2), and this tool will also be leveraged as a component of this feasibility clinical trial. Cardiovascular risk information from the artificial intelligence algorithm output will be placed alongside evidence-based suggestions for the patient’s care from the rules-based algorithm. The cardiovascular risk information and the evidence-based suggestions will be visually displayed side-by-side; this is the clinical decision aid, which is one of the most novel parts of this trial. Clinicians will meet with each patient and use the clinical decision aid to make recommendations for management based on these results plus their own judgement, taking into account each patient’s individual characteristics. Each patient will collaboratively decide with their clinician whether they will follow the medication use and imaging surveillance suggestions based on existing guidelines, expert recommendations, and medical society scientific statements will be created. We will then assess differences in medication use and imaging surveillance consistent with national guidelines and recommendations, as well as perceptions of the tool using surveys and focus groups.

**Table 1:** Patient baseline characteristics

*Sociodemographic Characteristics*	*Geocoding*
Age	County
Race	State
Sex	Zip
Marital status	
Employment status	
Ethnicity	
Language	
*Cardiovascular Conditions/Comorbidities*	*Tumor Characteristics*
Atrial Fibrillation	Diagnosis
Coronary Artery Disease	Site
Cardiomegaly	Type
Cardiomyopathy	Histology
Diabetes	Behavior
Hyperlipidemia	Stage
Heart Failure	Grade
Myocardial Infarction	Metastasis
Peripheral Artery Disease	Surgical margins
Stroke	
*Major Cardiovascular Medication Classes*	*Cancer Medication Classes*
ACE Inhibitors	Antineoplastic Antibiotics (anthracyclines)
Antianginals	Antineoplastic Enzyme Inhibitors
Angiotensin II Receptor Antagonists	Antineoplastic—Antibodies
Beta Blockers	Antineoplastic—Anti-HER2 Agents
Alpha–Beta Blockers	Antineoplastic—Angiogenesis Inhibitors
Alpha 2 Inhibitors	Antineoplastic—EGFR Inhibitors
Calcium Channel Blockers	Mitotic Inhibitors
Diuretics	Antineoplastic—Hormonal and Related Agents
Antihyperlipidemics	Antineoplastic—Immunomodulators
Antihypertensives	Chemotherapy Adjuncts
Antiarrhythmic	Antineoplastic—PDGFR-alpha Inhibitors
Anticoagulants	Antineoplastic—Hedgehog Pathway Inhibitors
Vasopressors	Antineoplastic—Cellular Immunotherapy
Mineralocorticoids	Alkylating agents
Phosphodiesterase Inhibitors	
Direct Renin Inhibitors	
Antidiabetic	

*ACE* Angiotensin-converting enzyme, *EGFR* Epidermal growth factor receptor, *HER2* Human epidermal growth factor receptor 2, *PDGFR* Platelet-derived growth factor receptor

**Table 2:** Patient outcomes/endpoints

**Primary Outcomes/Endpoints**
***Cardiovascular Imaging Recently Obtained***
*Cardiac Magnetic Resonance Imaging*
*Coronary Calcium Scan*
*Coronary Computed Tomography Angiography*
*Electrocardiogram ordered*
*Transthoracic echocardiogram*
***Cardiovascular Imaging Ordered***
*Cardiac Magnetic Resonance Imaging*
*Coronary Calcium Scan*
*Coronary Computed Tomography Angiography*
*Electrocardiogram ordered*
*Transthoracic echocardiogram ordered*
***Pre-Existing Cardiovascular-Related Medications***
*Patient already on ACE Inhibitor*
*Patient already on ARB*
*Patient already on Beta Blocker*
*Patient already on Statin*
*Patient already on Other Cardiovascular Medications*
*Patient already on Antidiabetic Medication*
***Cardiovascular-Related Medications Ordered***
*Prescription of ACE Inhibitor*
*Prescription of ARB*
*Prescription of Beta Blocker*
*Prescription of Statin*
*Prescription of Other Cardiovascular Medications*
*Prescription of Antidiabetic Medication*
**Secondary Outcomes/Endpoints**
*Survey Results*
*Focus Group Findings*
*Relevant Lab Testing Recently Obtained*
*Relevant Lab Testing Obtained*

*ACE* Angiotensin-converting enzyme, *ARB* Angiotensin receptor blocker

**Table 3:** Algorithm variables in the study. Used with permission [29]

Lab test (including demographic)	Echocardiographic
Sex	LVEF (left ventricular ejection fraction)
Race	Heart rate
Family history	BSA (body surface area)
Tobacco use	SBP (systolic blood pressure)
Alcohol use	DBP (diastolic blood pressure)
Diabetes	EDV (end-diastolic volume)
Hypertension	ESV (end-systolic volume)
Hyperlipidemia	LVEDVi (left ventricular end-diastolic volume index)
Peripheral edema	LVESVi (left ventricular end-systolic volume index)
Orthopnea	
Chest pain	
Shortness of breath	
Fatigue	
Age	
BMI (body mass index)	
eGFR (estimated glomerular filtration rate)	
RBC (red blood cell)	
Hematocrit	
MCHC (mean corpuscular hemoglobin concentration)	
MCV (mean corpuscular volume)	
MCH (mean corpuscular hemoglobin)	
Blood glucose	
Calcium	
Total protein	
Sodium	
Potassium	
Chloride	
Carbon dioxide	
WBC (white blood cell)	
Platelet	
Creatinine	
ALT (alanine aminotransferase)	
AST (aspartate aminotransferase)	
Albumin	
ALP (alkaline phosphatase)	
Bilirubin

Prior to implementing this protocol, the protocol, informed consent form, health insurance portability and accountability act authorization and any other information pertaining to participants will be approved by the Medical College of Wisconsin institutional review board.

### Study population

The overall study cohort dataset of > 4,000 individuals includes demographic, physiological, laboratory, medication, medical history, and outcomes data relevant to cardiovascular risk stratification in individuals with a history of cancer, as well as cardiovascular imaging reports [30]. This cohort will be queried to identify ≥ 200 adult cancer survivors (including ethnic/racial minorities) clinically considered to be at intermediate, high, or very high cardiovascular risk following cancer therapy determined imprecisely based on demographic and comorbidity information [3, 11, 31–34].

### Recruitment & randomization

From among these same ≥ 200 patients, our team will partner with the patients’ primary care providers (PCPs), hematologists, or oncologists to recruit and consent 200 adult cancer survivors for clinic visits and focus groups for the remainder of the study. The study team members will contact each potential patient to gauge their interest to participate in the study. Potential study participants who exhibit interest and/or agree to enroll in the study will be provided an information packet containing an informed consent form that offers a more in-depth description of the study and contact information if they are to have additional questions. Those who do not express interest in the study will be noted and no longer contacted. Study participants will be randomly distributed into either the Clinical Decision Aid Group (where *N* = 100 patients will use the clinical decision aid in addition to current practice) or the Control Group (where *N* = 100 patients will only have access to current practice) based on the following stratification factors: sex and race/ethnicity. In collaboration with our biostatistician, patients will be randomly distributed into one of these two arms based on the following stratification factors: sex (2: male, female) and race/ethnicity (3: White, Black, other).

### Clinical visits and clinical decision aid intervention

**Fig. 3:**
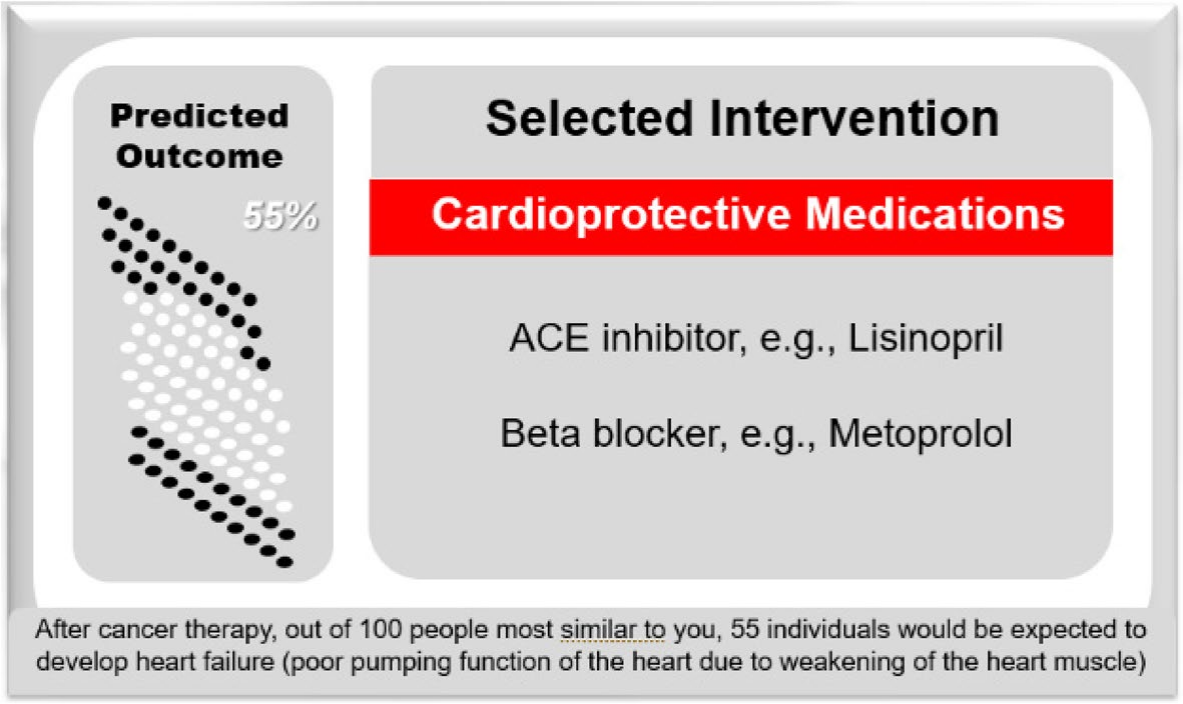
Artificial Intelligence-Powered Clinical Decision Aid Mockup. The visual interface will continually be iterated and improved based on patient and clinician feedback during the study and beyond; additional components of the mockup illustrate the explainability and transparency of the algorithms and models employed in the clinical decision aid

Patients in the study will meet with study cardiologists in either a virtual or in-person clinically indicated visit, with technical support and training provided by the study team as needed. For those in the Clinical Decision Aid Group, their personalized risk output from the artificial intelligence algorithms will be organized in a meaningful and user-friendly way, using customized pictographs visually representing personalized and precise patient risk in the clinical decision aid (Fig. 3). This will be juxtaposed with suggestions for medication use and imaging based on existing guidelines, expert recommendations, and society scientific statements (from Fig. 2).

The clinical decision aid will be made available via a simple web-based interface hosted online at the Cardiology Oncology Innovation Network (CardioOncCOIN.Org). A link to the clinical decision aid webpage interface will be placed in each patient’s electronic health record. Study physicians can click on this link in the patient’s chart to access the webpage. In this way, the webpage interface will be independent of the particular electronic health record. We will also initiate efforts to have the same clinical decision aid integrated into Epic via the Epic App Orchard. If we are able to achieve this, then the study physician will also be able to access the clinical decision aid in this way.

In both study arms, video and audio may be recorded throughout the clinical visit for use in subsequent objective analysis of shared decision-making, the collaborative process by which health care choices are made by the patient with guidance from a health care professional [35]. Shared decision-making will be scored by study staff using the OPTION5 scale [36–38]. Study participants will be asked to complete established Likert scale surveys that reflect their personal health perceptions, decisions, and behaviors.

### Focus groups

As part of an exploratory study, a total of 20 patients in the Clinical Decision Aid Group and all the study physicians will attend two patient or physician focus group sessions, respectively. The focus groups will discuss the virtual clinical decision aid interface and how to improve it. Treating physicians will meet separately from patients in their own focus groups. The first will be held a week after the initial visit and the second held a week after a clinically indicated follow-up visit when applicable. Focus groups will be run by our qualitative research collaborators who will facilitate discussions about the user experience, to obtain critical feedback on the virtual clinical decision aid interface. The expectations for these focus group sessions are to help our study team further develop and improve the clinical decision aid interface to fit the needs of patients and their clinicians.

### Follow-up clinic visits

Follow-up clinic visits may be pursued as clinically indicated. Patients in the Clinical Decision Aid Group may again use the clinical decision aid, which would be an updated version incorporating some of the focus group feedback and suggestions. In addition, study participants in the Control Group will receive their risk group information at the end of the study if desired and can review again at that time with their clinicians the preventive steps they have taken for heart health.

### Primary endpoints

Chart reviews will also be completed for all patients to assess medication use and imaging patterns in accordance with cardio-oncology recommendations at three and six months after the baseline clinic visit, as the primary endpoints.

If the patient chooses not to or is unable to pursue the recommendations suggested by the clinical decision aid as discussed by the physician, this would be noted in the chart. Therefore, if the physician offers the recommendation, this will be considered consistent with the society statements, even if the patient chooses not to follow the recommendation. An adjudication committee of 3 people may be established to decide what is considered consistent with evidence-based recommendations. If 2 of 3 people agree, that would be considered consistent with evidence-based recommendations.

Success will be determined by ≥ 85% of clinic visits using the clinical decision aid resulting in pursuit of medication use and cardiac imaging surveillance patterns commensurate with recommendations specific to cancer survivors, with overall rates the same or higher than the group without the clinical decision aid, as well as overall favorable patient and physician focus group comments and survey responses.

### Secondary endpoints

Survey responses will be reviewed to determine the impact of the use of the clinical decision aid, in addition to current practice, on cancer survivors’ perceptions of the tool and heart health-related behaviors. The percentages of favorable survey responses and scoring tool results in the patient group using the clinical decision aid will be compared to results from the patient group that does not use the clinical decision aid. A high score will indicate favorable responses for a particular survey, demonstrating favorable perception of the tool regarding the survey topic. We anticipate ≥ 80% of survey questions scored favorably by the group of patients who use the clinical decision aid, with scores the same or higher than the group without the clinical decision aid [39].

Clinically indicated lab values such as troponin, NT-pro-BNP, potassium, and lipid panel, along with BMI, will also be reviewed.

### Exploratory endpoints

Overall favorability of the clinical decision aid will be evaluated using patient and physician focus group comments collated qualitatively. Cancer survivors and physicians will be asked to comment on particular features of the clinical decision aid that contribute to improving usability, understandability, and visual acceptability of the clinical decision aid. This will help us identify ways in which the clinical decision aid tool can be further improved for use by both patients and clinicians.

### Statistical methods

#### Sample size and power analysis

Assuming 40% consistency with evidence-based recommendations at baseline for both groups [18, 20, 40], we will consider clinical significance at a 50% increase from baseline, which corresponds to 60% consistency with the recommendations. To go from 40% at baseline to 60% in the intervention arm of the trial (i.e., Clinical Decision Aid group) with 80% power requires 97 patients in each group, or 194 total patients. We may be powered to detect such a clinically reasonable change in the primary endpoint. Although we may be powered for this modest change, we could also consider a greater increase from baseline. To go from 40 to 85% with 80% power requires 17 patients in each group, or 34 total patients. We would also be powered for this, but this increase may not be reasonably obtainable.

In this feasibility study, the total number of patients will be limited to 200. One cohort of 100 patients will have clinic visits with current practice plus the clinical decision aid. The other cohort of 100 patients will have clinic visits with current practice alone. This should allow for capturing a variety of cancers and include ethnic minorities in each group.

### Data analysis

We will compare the following measurements between the Clinical Decision Aid Group and the current practice only Control Group: baseline characteristics, perceptions and attitudes toward decision-making in the clinic visit, medication initiation (e.g., statins or beta blockers for cardioprotection), cardiovascular lab testing obtained, physical activity pursued (based on survey responses), cardiovascular imaging tests ordered or recommended for surveillance. We will also analyze data using qualitative descriptive statistics for focus group results from patients and clinicians in the Clinical Decision Aid Group.

Scores will be calculated for individual survey questions, with a higher number indicating a more favorable response. The randomization groups will be considered as the independent variables and survey scores, as well as short-term outcome measures, such as cardiovascular medication initiation and imaging parameters, and lab test results if applicable, as the dependent variables. Survey scores, imaging parameters, medication use, and lab tests if applicable, will be compared between the group of patients that use the clinical decision aid (Clinical Decision Aid Group) and the group of patients that do not use the clinical decision aid (Control Group) at single visits (baseline, three months, six months). Additionally, survey scores for information seeking and sharing, as well as imaging parameters and medication use, and lab tests if indicated, will also be compared between the Clinical Decision Aid Group and the Control Group between visits. Thus, changes over time in the Information Seeking and Sharing survey responses, imaging parameters, medication use, and lab tests if results applicable, will be investigated. We will assess whether survey responses, imaging parameters, medication use, and lab tests if applicable, differ by study group (Clinical Decision Aid Group versus Control Group) or by risk-stratified patient similarity cluster. We may also pursue subgroup analyses to compare clinical decision aid and Control Group results within each patient similarity risk cluster, between races, or by sex.

Simple group comparisons will be made using the chi-square or Fisher’s exact test as appropriate for binary survey score variables. Logistic regression will also be used to estimate the effect of the randomized group on the binary score for each survey question, to allow us to adjust data for the following baseline socioeconomic demographics: age, sex, and level of education if known. These demographic characteristics will also be investigated as potential predictors using multivariate analyses, for survey questions or short-term outcomes with results significantly different between the two randomized groups. Continuous variables will be expressed as mean with standard deviation, whereas dichotomous variables will be expressed as percentages. For continuous characteristics or outcomes, Wilcoxon rank sum test or two-sample t-test will be used as appropriate. Statistical data will be expressed as odds ratio with confidence interval or mean with standard error. Statistical significance will be accepted as *P*-value of < 0.05.

## Discussion

In this clinical trial, we will assess whether the use of a novel clinical decision aid tool will improve the extent to which cancer survivors’ cardiovascular medication use and imaging surveillance pursued align with the current cardio-oncology guidelines, expert recommendations, and society scientific statements. This feasibility study will provide information regarding personalized care using an innovative clinical decision aid for cancer survivors at risk for cardiovascular toxicities from cancer therapies.

The study population will include cancer survivors facing a range of cardiovascular diseases and risks. With the introduction of a pictograph generated by the clinical decision tool, we hope to provide patients with customized and precise cardiovascular risk information. Concurrently, suggestions for care are provided based on existing guidelines, expert recommendations, and medical society scientific statements. Together with current practice they receive from their cardiologist, these measures may further enhance their care quality. Patient and physician attitudes, decisions, and behaviors, with short-term outcomes (medication use and imaging utilization) documented from this study, may provide valuable guidance and tools for oncologists, cardiologists, informaticians, and administrators tasked with improving prediction and care of cancer survivors in cardio-oncology.

New successful cancer therapies have resulted in a greater number of survivors, but also have increased cardiovascular disease risks [23, 31, 32, 41]. Therefore, the challenge remains of supporting physicians and cancer survivors in adopting standard-of-care recommendations into clinical practice, especially for those cancer survivors at highest cardiovascular risk [18, 20, 40]. The use of artificial intelligence has the potential to transform personalized risk assessment options for our patients [42]. By enhancing clinical assessment with AI prediction algorithms, clinicians might feel more confident with individualized risk prediction and pursue more aggressive guideline-based management.

Simultaneously, care suggestions based on current guidelines, expert consensus, and medical society scientific statements that guide standard-of-care practices are provided in the clinical decision aid. This study therefore carries limited risk, since patients will collaborate with their physician to develop a plan of care that adheres to the best cardiology practices. The clinical decision aid may affect a subject’s cardioprotective decisions and behaviors, including standard medication initiation. Based on previous studies, we anticipate that the use of artificial intelligence-powered and other personalized clinical decision support/aid tools combined with electronic health record data may improve patient outcomes, notably for prevention and early detection of cardiovascular disease (e.g., by 35–75%) [43, 44].

In conclusion, this study will assess whether an artificial intelligence-powered clinical decision aid that presents care recommendations based on current guidelines, expert recommendations, and medical society scientific statements will favorably improve cardioprotective medication use and cardiac imaging utilization for cancer survivors. The results of the study may have implications for digital transformation in the cardiovascular care of cancer survivors.

## Data Availability

Data can be provided upon request.
